# Translation and validation of the reaction to impairment and disability inventory in a chinese ccontext

**DOI:** 10.1192/j.eurpsy.2021.2095

**Published:** 2021-08-13

**Authors:** A. Siu

**Affiliations:** Rehabilitation Sciences, The Hong Kong Polytechnic University, Hunghom, KOwloon, Hong Kong PRC

**Keywords:** Chronic illness and disability, emotional adjustment, Psychosocial adaptation, Chinese

## Abstract

**Introduction:**

People with chronic illness and disabilities (CID) often need to adjust to changes in self-concept, cope with their grief from the loss of functional abilities, and to “live with the illness”. Emotional adjustment to disabilities is a major challenge in rehabilitation, but there is no validated Chinese instrument for assessing psychosocial adaptation of people with CID.

**Objectives:**

This study translated the Reaction to Impairment and Disability Inventory (RIDI) into Chinese and validated the Chinese version (C-RIDI), for assessing emotional adjustment in people with CID. We examined the factor structure, internal consistency, convergent validity, and criterion-related validity of the C-RIDI.

**Methods:**

We conducted a survey of people with CID who were recruited from community-rehabilitation settings and self-help groups (n = 244). The research questionnaire collected demographic information, illness-related variables, the C-RIDI, and measures of resilience and well-being.

**Results:**

The C-RIDI has good content validity and no major changes to the translated items were needed for the use with Chinese population. For factor structure, we replicated the results of Livneh, Martz, & Boder (2006). The C-RIDI has two second-order factors of adaptive and non-adaptive scales, which interact with the two denial subscales. Internal consistency of the subscales is satisfactory except for the 3-item denial subscales. Correlations of the C-RIDI subscales with illness-related variables, resilience, and mental well-being are consistent with our hypotheses and provide support for the convergent and criterion-related validity of the scale.

**Conclusions:**

The C-RIDI has satisfactory psychometric properties. The study results support its internal consistency, convergent validity, criterion-related validity, and factorial validity.

**Disclosure:**

No significant relationships.

